# Fibrin Scaffold Incorporating Platelet Lysate Enhance Follicle Survival and Angiogenesis in Cryopreserved Preantral Follicle Transplantation

**DOI:** 10.31661/gmj.v9i0.1558

**Published:** 2020-07-08

**Authors:** Alireza Rajabzadeh, Fatemeh Jahanpeyma, Ali Talebi, Faezeh Moradi, Amir Ali Hamidieh, Hussein Eimani

**Affiliations:** ^1^Department of Tissue Engineering and Applied Cell Science, School of Advanced Technologies in Medicine, Tehran University of Medical Sciences, Tehran, Iran; ^2^Department of Medical Biotechnology, Faculty of Medical Sciences, Tarbiat Modares University, Tehran, Iran; ^3^School of Medicine, Shahroud University of Medical Sciences, Shahroud, Iran; ^4^Department of Tissue Engineering, Faculty of Medical Sciences, Tarbiat Modares University, Tehran, Iran; ^5^Pediatric Stem Cell Transplant Department, Children’s Medical center, Tehran University of Medical Sciences, Tehran, Iran; ^6^Department of Embryology, Reproductive Biomedicine Research Center, Royan Institute for Reproductive Biomedicine, ACECR, Tehran, Iran

**Keywords:** Ovarian Follicle, Cryopreservation, Tissue Scaffolds, Fibrin

## Abstract

**Background:**

Transplantation of cryopreserved follicles can be regarded as a promising strategy for preserving fertility in cancer patients under chemotherapy and radiotherapy by reducing the risk of cancer recurrence. The present study aimed to evaluate whether fibrin hydrogel supplemented with platelet lysate (PL) could be applied to enhance follicular survival, growth, and angiogenesis in cryopreserved preantral follicle grafts.

**Materials and Methods:**

Preantral follicles were extracted from 15 four-week-old NMRI mice, cryopreserved by cryotop method, and encapsulated in fibrin-platelet lysate for subsequent heterotopic (subcutaneous) auto-transplantation into the neck. Transplants were assessed in three groups including fresh follicles in fibrin-15%PL, cryopreserved follicles in fibrin-15%PL, and cryopreserved follicles in fibrin-0% PL. Two weeks after transplantation, histological, and immunohistochemistry (CD31) analysis were applied to evaluate follicle morphology, survival rate, and vascular formation, respectively.

**Results:**

Based on the results, fibrin-15% PL significantly increased neovascularization and survival rate (SR) both in cryopreserved (SR=66.96%) and fresh follicle (SR=90.8%) grafts, compared to PL-less fibrin cryopreserved transplants (SR=28.46%). The grafts supplemented with PL included a significantly higher percentage of preantral and antral follicles. Also, no significant difference was observed in the percentage of preantral follicles between cryopreserved and fresh grafts of fibrin-15% PL. However, a significantly lower (P=0.03) percentage of follicles (23.37%) increased to the antral stage in cryopreserved grafts of fibrin-15%PL, compared to fresh grafts (35.01%).

**Conclusion:**

The findings demonstrated that fibrin-PL matrix could be a promising strategy to improve cryopreserved follicle transplantation and preserve fertility in cancer patients at the risk of ovarian failure.

## Introduction

Recent advances in cancer treatment have markedly improved survival rates in many cancers, although chemotherapeutic regimens and radiation therapy may induce loss of ovarian function and infertility in pre-menopausal patients. In particular, alkylating agents, platinum-based drugs, and ionizing irradiation to the pelvis are known to cause gonadotoxicity [1,2]. During the last 20 years, several options for fertility preservation have been proposed, although the choice of best technique depends on the patient’s age, gender, relationship status, cancer type, and future reproductive goals. The established procedures for preserving fertility include oocyte, embryo cryopreservation, and ovarian transplantation. However, some experimental options were reported in development such as ovarian cryopreservation and transplantation, oocyte in vitro maturation, and in vitro growth of primordial follicles, some of which were proven to be successful [3]. Among the established approaches, cryopreservation and transplantation of ovarian tissue have become a widely used procedure for restoring ovarian function and fertility in cancer patients. However, there remains a significant risk of the presence of malignant cells in cryopreserved tissues, which may cause a recurrence of the primary disease [2]. In this regard, re-implanting ovarian tissue in these patients is not recommended since some types of cancer include leukemia and breast cancer may lead to metastasis to the ovaries [4]. Isolation and transplantation of follicles can reduce the risk of reseeding malignant cells because the surrounding basement membrane in ovarian follicles separates them from stroma tissues, nerves, and blood vessels [1]. The ovary contains follicles at multiple stages of development include primordial, primary, secondary, preantral, antral, and pre-ovulatory follicles [5]. In mammals, a large number of follicles are available in the primordial and preantral developmental stage. Preantral follicles, as reported by Itoh *et al*., can be a potential source of genetic material and their oocytes can be implemented in reproducing animal, as well as conserving species at the risk of extinction and transgenesis [6]. Further, culture systems for isolated preantral follicles have been successfully established in mice, bovine, porcine, caprine, as well as in human [6-11]. Although several reports have already documented the successful implantation of primordial and preantral follicles [2,4,7], no approach has been widely implemented in this regard. Compared to the transplantation of cryopreserved ovary, cryopreservation and implantation of isolated follicles is less complicated, which may be related to the tissues associated with the complex structure of ovarian and harboring compact stroma, as well as different types of cells which make the cryopreservation process complicated [5,12]. Thus, a biocompatible and biodegradable scaffold is required to maintain the three-dimensional structure of isolated follicles and provide support for their development [1]. Providing an environment for cell differentiation and proliferation, vessel recruitment, cell invasion, and accordingly, follicle survival and growth is considered as the main advantage of this approach [1,4]. Fibrin has been utilized as a cell-embedding material for tissue engineering of cartilage and bone [13], transplantation of mesenchymal cells [14], human keratinocytes [15], limbal stem cells [16], inducible pluripotent stem cells (iPS) [17]. Bone marrow-derived mononuclear cell (BMNC) [18], and recently ovarian follicles [1]. This matrix was proven to be biodegradable and biocompatible [12], which may provide sufficient diffusion capacity for proliferative cells [13]. Successful transplantation depends on neovascularization, although a delay in vascularization may result in creating ischemia and loss of grafted follicles [12]. Platelet concentrates (PC) [19] including platelet lysate (PL), prepared by freeze-thawing the PC, could support proliferation, wound healing, tissue, and vascular regeneration [20,21]. Various growth factors which platelets store in their α-granules including vascular endothelial growth factor (VEGF), insulin-like growth factor (IGF), platelet-derived growth factors (PDGF) and basic fibroblast growth factor (bFGF) are involved in vasculogenesis and survival of endothelial cells [12,20]. In a recent study, four fibrin-PL formulations containing different concentrations of PL were investigated, and the formulation with 15% PL promoted follicular survival and growth [12]. By considering the research mentioned above, the present study aimed to evaluate the effect of biodegradable fibrin scaffold supplemented with 15% PL on the survival and growth of cryopreserved ovarian follicle grafts. To this end, the preantral follicles encapsulated in the biomaterial scaffold (fibrin+15% PL) were transplanted into mice following vitrification and thawing. Then, the grafted follicles were assessed for cell survival, maturation, oocyte generation, and vasculogenesis to evaluate their ability to preserve fertility and extend transplantation-based options.

## Materials and Methods

###  Experimental Design

In the present study, follicular growth and survival were evaluated after vitrification and thawing in Naval Medical Research Institute (NMRI) mice (n=15) **. **The guidelines for animal welfare were approved by the local Ethics Committee at Royan Institute (approval code: k/90/004). Four-week-old female NMRI mice from the Pasteur Institute (Tehran, Iran) were used in the present study. Also, the mice were housed in pairs in the rooms maintained at 20-25 ˚C, 50% humidity, and with a 12-h light/12-h dark cycle. Left ovary of each mouse was excised to isolate preantral follicles. Further, a total of 336 follicles were encapsulated in fibrin hydrogel containing 15% PL and evaluated in 15 independent experiments following cryopreservation and auto-transplantation. Accordingly, the obtained results from implantation of cryopreserved follicles were compared to the fresh follicles encapsulated in the same biomaterial matrix (n=320), as well as to the grafts of cryopreserved follicles in PL-less fibrin (n=311). In the next stage, the encapsulated follicles were transplanted under the cervical fascia in the same mouse. Then, the encapsulated follicles which were not grafted were assessed by viability test to measure follicular viability before transplantation. After 14 days of autotransplantation, the transplants were analyzed for follicular survival and growth. Finally, the process of angiogenesis was evaluated by immunohistochemistry.

###  Ovariectomy Procedure

First, the NMRI mice were anesthetized by intraperitoneal injection of ketamine (96 mg/kg, Sigma, Germany) and xylazine (256 mg/kg, 2% Alfasan Sigma, Germany). Then, each mouse was ovariectomized through a single dorsal transverse skin incision, allowing sufficient access to the left abdomen. In addition, the ovarian bursa containing the ovary was excised and placed in 200 µL alpha-minimal essential medium (α –MEM, Gibco-Invitrogen, Germany) containing 10% fetal bovine serum (FBS, Sigma Aldrich, USA). The MEM+10% FBS droplets were previously covered by mineral oil and incubated for 24 h in the CO_2_ incubator. Further, the surrounding tissues were rinsed and removed by using surgical scissors. Finally, the peritoneum was closed with an interrupted stitch of the absorbable suture.

###  Isolation of Preantral Follicles from Mouse Ovaries

The preantral follicles were mechanically isolated as previously described by Cortvrindt *et al*. [Cortvrindt, 1996 #6]. Briefly, the follicles in the α-MEM+10% FBS medium were collected using a 29-gauge needle attached to a 1 ml syringe under a stereo-microscope. The circular preantral follicles including 2 to 3 layers of granulosa cells and a central oocyte were considered to be morphologically normal for subsequent experiments. In the next step, follicles diameters were measured along two axes with a micrometer in the eyepiece of an inverted microscope (Olympus, CKX41, Japan). Finally, the diameter was estimated as the average of vertical and horizontal measurements.

###  Preparation of PC

First, 350-400 ml of human umbilical cord blood was centrifuged for 22 min at 300 g (37 ˚C) to obtain platelet-rich plasma (PRP). After initial centrifugation, the harvested PRP was re-centrifuged at room temperature at approximately 300 g for 30 min to obtain PC [19]. The number of platelets in each sample was estimated by multiplying the blood or plasma volume by the platelet count. The samples of approximately 10^9^ platelets/ml were selected and stored at ˗70 ˚C until required. In order to obtain platelet-released growth factors, the frozen PC was thawed and centrifuged for 30 min at 3000 g at 4 ˚C to remove the platelet debris.

###  Vitrification and Thawing 

The follicles that were already placed in the α-MEM+10% FBS were vitrified with a slight modification according to the protocol described by Hasegawa *et al*.[22]. Then, the isolated preantral follicles (100-130 µm) were suspended in an equilibrium solution containing 7.5% ethylene glycol and 7.5% dimethyl sulfoxide in α-MEM medium (PH=7.5) including 20% FBS for 3 min at the room temperature. The follicles were instantly transferred to the vitrification solution including 15% dimethyl sulfoxide, 15% ethylene glycol, and 0.5 M sucrose in α-MEM+ 20% FBS medium for 1 min at the room temperature. Also, 3-6 follicles were loaded on a Cryotop® SC (Kitazato, Japan) in a low volume (<0.1 µl) of the vitrification solution, and plunged immediately in liquid nitrogen. For warming, the cryotops were directly transferred from liquid nitrogen into the α-MEM medium composed of 20% FBS, 6 µg/ml penicillin, 5 µg/ml streptomycin and 1.0 M sucrose at 37 ˚C for 1min. Then, the follicles detached from the cryotop were placed in 0.5 M sucrose solution in α-MEM medium supplemented with 10% FBS, 6 µg/ml penicillin, and 5 µg/ml streptomycin for 3-7 min. Subsequently, they were re-suspended in α-MEM+ 10% FBS medium supplemented with the antibiotics as described earlier in this section (2×5 min). The follicles were incubated in the final medium for 1 h to restore the initial morphology.

###  Encapsulation of Follicles in PL-Fibrin Hydrogel

**Figure 1:**
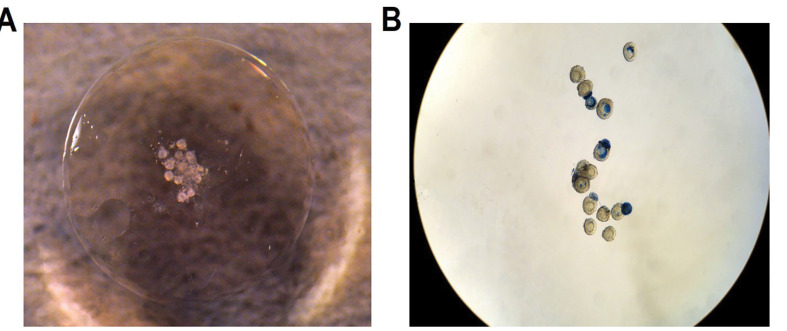
**A:** Fibrin scaffold seeded with isolated preantral follicles. **B:** Live/dead assay of encapsulated follicles in fibrin hydrogel.

First, thrombin and fibrinogen were obtained from the Cell Science Research Center of Royan Institute (Tehran, Iran). Then, fibrin glue/hydrogel was prepared as previously described by Luyckx *et al.* [1], but with a slight modification. Also, a droplet of 8.5 µL thrombin (4 IU/ml) and 2.5 µL PL were placed on a petri dish, and 21 follicles (vitrification-thawed) were carefully suspended in the thrombin droplet. Further, 4.25 µL of fibrinogen (25 mg/ml) solution was added to this droplet (2:1) and incubated in a CO_2_ incubator at 37 ˚C for 5 min. The hydrogel was ready for implantation to the host mother after 2 min (Figure-1A).

###  Viability Testing 

 After vitrification and thawing, follicular viability was assessed to determine any deleterious effect of follicle isolation process and freeze-thawing on the quality of prenatal follicles. To this end, dead and live cells were identified using methylene blue (Sigma, Germany) staining. The follicles were cultured in the α-MEM supplemented with 10 mIU/ml Follicular Stimulation Hormone (FSH, Merk, Germany), 1 mIU/ml Luteinizing Hormone (LH, Merk, Germany), 1% Insulin Transferrin Selenium (ITS, Gibco, UK) and 5% FBS (PH=7.2-7.4) for 24 hours. Then, a droplet of 100 µL culture suspension containing follicles was placed on a petri dish, and 40 µL of methylene blue was added to the droplet. Further, the follicles were incubated with the dye at 37 ˚C for 5 min so that methylene blue can be easily penetrated cells. Finally, the dead follicles were stained blue, and the live ones were not stained.

###  Auto-Transplantation Procedure

To graft the fibrin hydrogels supplemented with 15% PL and containing preantral follicles, the mice hair on the neck region was removed using a chemical depilator. Each mouse was anesthetized using the same protocol as for the ovariectomy procedure. Through a single dorsal transverse incision, a subcutaneous pocket was created in the posterior area of the neck. The fibrin hydrogels were gently placed into the pocket, and the skin was closed with polyethylene suture 6 (Ethicon, USA). Then, the mice were recovered and housed under standard conditions. After two weeks, the mice were euthanized by CO_2_ asphyxiation, and the follicles were fixed in bouin’s fixative.

###  Histological Analysis

In order to evaluate the grafted follicles, histological analysis was accomplished on grafted fibrin-encapsulated follicles. After fixation in bouin’s fixative, the grafts were dehydrated, embedded in paraffin, and cut into 6 µm serial sections. Then, every fourth section was stained with hematoxylin-eosin (Merck, Darmstadt, Germany) for histological analysis. Also, the other sections were kept for immunohistochemical analysis. The morphologically normal follicles were counted based on the method described by Israeali *et al*. [23]. The quality of follicular cells was evaluated based on membrane integrity, observation of pyknotic bodies, oocyte integrity, and cellular density [1]. Further, the follicles were classified according to the stage as primordial, primary, preantral, and antral under a light microscope at 40× and 100× magnification. Furthermore, the primordial follicles were distinguished by oocytes lacking a zona pellucida, and surrounded by one layer of granulosa cells, primary follicles by a layer of cuboidal granulosa cells, preantral by two or more layers of granulosa cells lacking antrum, and the antral follicles by the presence of an antral cavity. Then, the oocytes were screened for signs of atresia including contraction and clumping related to the chromatin, eosinophilia of ooplasm, and membrane damage and wrinkle formation [12,24]. Based on the described criteria, the grafted follicles were classified as normal and degenerated. The percentage of normal follicles in the histological slices was reported as the survival rate in the result section.

###  Immunohistochemistry

 The endothelial cells were assessed by CD31 immunostaining to analyze angiogenesis in the grafted follicles. The CD31 platelet marker or endothelial cell adhesion (PECAM-1) is a 130-kD membrane glycoprotein, which is expressed on platelets and most leukocytes. Initially, the slides were deparaffinized with xylol solution (2×10 min) and rehydrated in graded series of ethanol (100, 90, 70, and 50%) and deionized water (3 min each). In order to retrieve the antigens, the sections were incubated in a solution including 1% calcium chloride and 0.5% trypsin for 20 min at 37 ˚C and 10 min at the room temperature. The slides were washed in PBS buffer containing 0.05% Tween 20 (2×10 min) and incubated for 1 h (37 ˚C) with goat serum to block nonspecific binding sites before adding rat-anti-mouse CD31 antibody. The slides were subsequently washed with PBS-Tween 0.05% for 2×5 min followed by incubation for 2 h at the room temperature with rat anti-mouse CD31 antibody (1:250 dilution). After washing with PBS-Tween 0.05% (3×15 min), the slides were incubated for 1 h at 37 ˚C with rabbit anti-rat antibody (1:200 dilution). Then, they were thoroughly washed in PBS-Tween 0.05% (2×7 min) and the cell nuclei were stained with DAPI reagent for 1 h. In the next procedure, the slides were mounted with DPX neutral mounting medium (Prosan, Merelbeke, Belgium) and observed under the fluorescent microscope. The total vessel surface area was measured using ImageJ 1.52h, free image processing software (ImageJ, National Institute of Health, Bethesda, Maryland, USA).

###  Statistical Analysis

 The data, expressed as mean ± standard error of mean (SEM), were analyzed by ANOVA. Tukey’s Honest Significant Differences Post hoc test was used for checking the homogeneity of variances, and Dunnett Post Hoc was used for heterogeneity. The analysis was conducted by GraphPad Prism version 5 (GraphPad Software, CA, USA). The probability of P≤0.05 was considered as statistically significant.

## Results

###  Follicle Viability Following Vitrification and Thawing

 A total of 149 preantral follicles were assessed for viability following cryotop vitrification. The live/dead viability assay was performed by using methylene blue staining 24 h after follicular culture (Figure-1B), among which 91.28% (136/149) were live follicles, and 8.72% (13/149) were damaged or dead follicles.

###  Evaluation and Histological Analysis of Follicles After Transplantation

**Figure 2:**
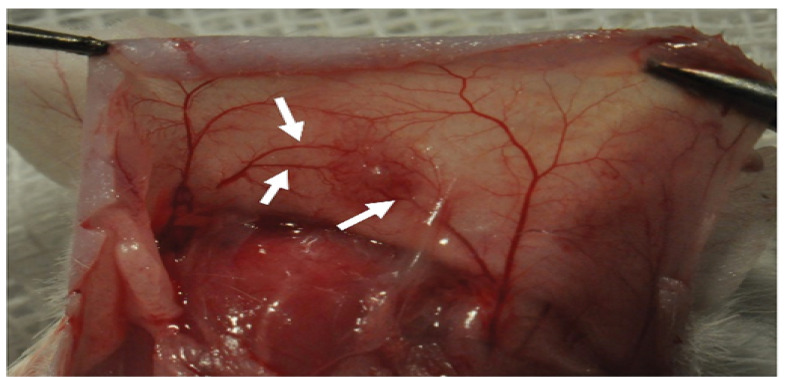
After two weeks of auto-transplantation, angiogenesis was directed toward the graft. The arrows are pointing to the branching graft capillaries around and through the graft.

**Figure 3:**
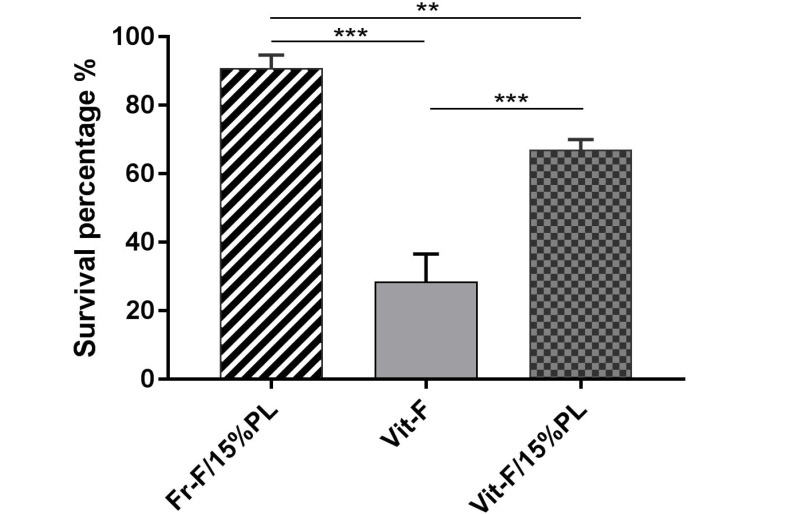
The survival rate of grafted vitrified and fresh follicles encapsulated in fibrin hydrogel with or without platelet lysate (PL). Each vertical bar represents the mean ±SD. **P=0.05, ***P<0.005.

**Figure 4:**
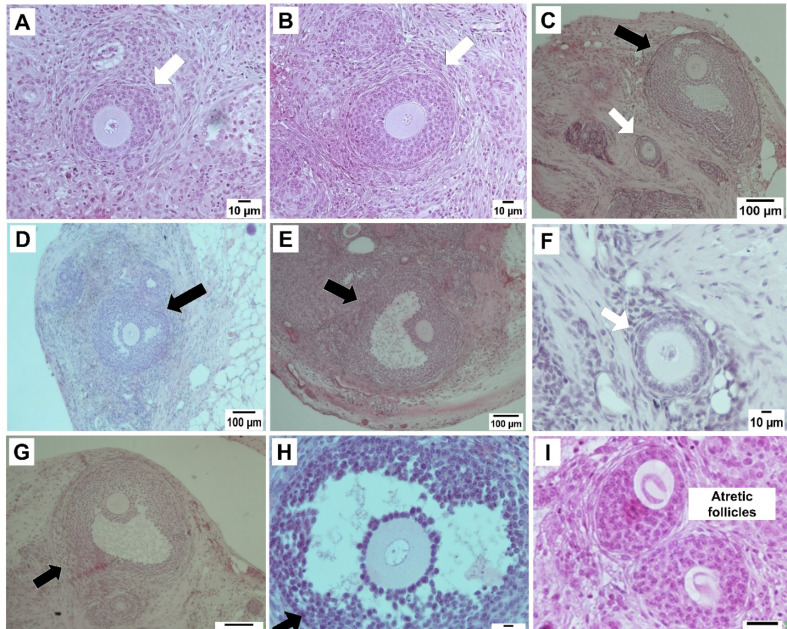
Hematoxylin-eosin staining of histological sections of murine ovarian follicles auto-grafted in fibrin-PL into the subcutaneous layer of 14-week mice. Normal preantral (white arrows) and antral follicles (black arrows) were observed in vitrified fibrin-15% PL ( **B**, **G**, **H**), vitrified PL-less fibrin ( **D**, **F**), and fresh fibrin-15%PL ( **A**, **C**, **E**) grafts after two weeks of transplantation. Follicle atresia was seen in some sections ( **I**).

**Figure 5:**
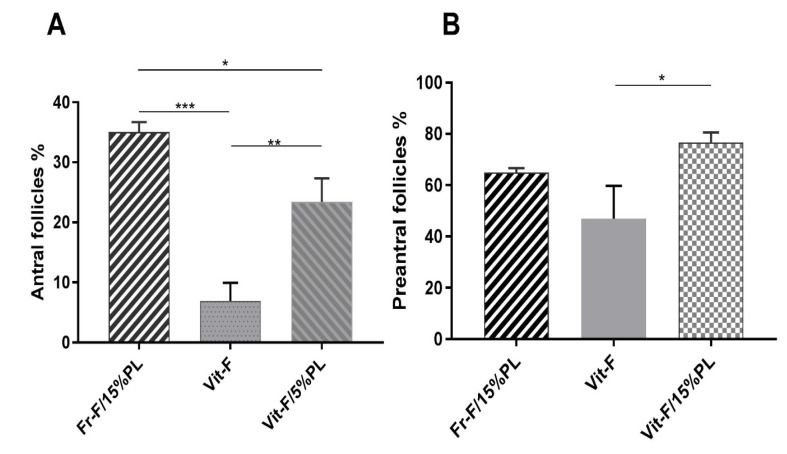
The percentage of preantral ( **A**) and antral ( **B**) follicles found in fresh follicles+ 15%PL, vitrified follicles+ 15%PL, and vitrified follicles -0%PL grafts. Each vertical bar represents the mean ±SD. *P˂0.02, **P=0.002, ***P<0.001.

Fifteen NMRI mice were successfully ovariectomized, and the isolated preantral follicles were auto-transplanted after encapsulation. Then, all of the grafts were retrieved 14 days after implantation surgery, while a vascular network surrounding the graft was macroscopically visible. In line with the results of the previous study, the grafts containing PL led to higher levels of branching vascular support, compared to the grafts without PL (Figure-2) [12]. According to the pre-described histological criteria, a survival rate of 90.8% was obtained following the transplantation of fresh follicles encapsulated in fibrin-15% PL. However, the implantation of cryopreserved follicles encapsulated in the same matrix led to a survival rate of 66.96%. In other words, a significant decrease (P=0.005) was observed in the survival rate of follicles after vitrification and thawing. Besides, using a PL-less fibrin scaffold for transplanting cryopreserved follicles resulted in lowering the survival rate significantly (28.46%), compared to vitrified fibrin+15% PL and fresh fibrin-15%PL groups (P<0.001, Figure-3). The results confirmed the significant effect of PL to improve the procedure of follicle transplantation, ischemia reduction, and follicle loss. Further, the follicles were morphologically examined in both cryopreserved and non-cryopreserved tissues. As shown in Figure-4, multiple growing follicles in preantral and antral stages were observed in the grafts from all recipient mice. Furthermore, preantral follicles were morphologically distinguished by observing the rounded structures containing an intact oocyte surrounded by two or more layers of granulosa cells (Figure-4). Additionally, antral follicles were reported as spherical structures containing a fluid-filled antral cavity surrounded by multiple layers of granulosa cells and few theca cells. In addition, the oocyte in these cells contained a large nucleus, which was surrounded by an intact zona pellucida (Figure-4C, D and E). Meanwhile, some sections included atretic follicles distinguished by observing clumped chromatin, membrane damage, and eosinophilia of ooplasm (Figure-4F) [12,24]. As shown in Figures-5A and B, no significant difference was observed in the percentage of preantral follicles between fresh and cryopreserved groups in fibrin-15%PL, although the percentage of antral follicles in cryopreserved fibrin-15%PL grafts was 66.7% lower than fresh fibrin-15%PL grafts (P=0.03). Further, the percentage of antral follicles in the cryopreserved fibrin-0%PL group (6.9%) was significantly lower than that of fresh fibrin-15%PL (P<0.001) and cryopreserved fibrin-15% PL groups (P=0.002). However, no significant difference was reported between the percentage of preantral follicles in vitrified PL-less fibrin and vitrified fibrin-15% PL grafts, while 61% less preantral follicles were observed in follicle grafts encapsulated in PL-less fibrin scaffold (P=0.02), compared to the fresh fibrin-15% PL group.

###  Evaluation of Revascularization

**Figure 6:**
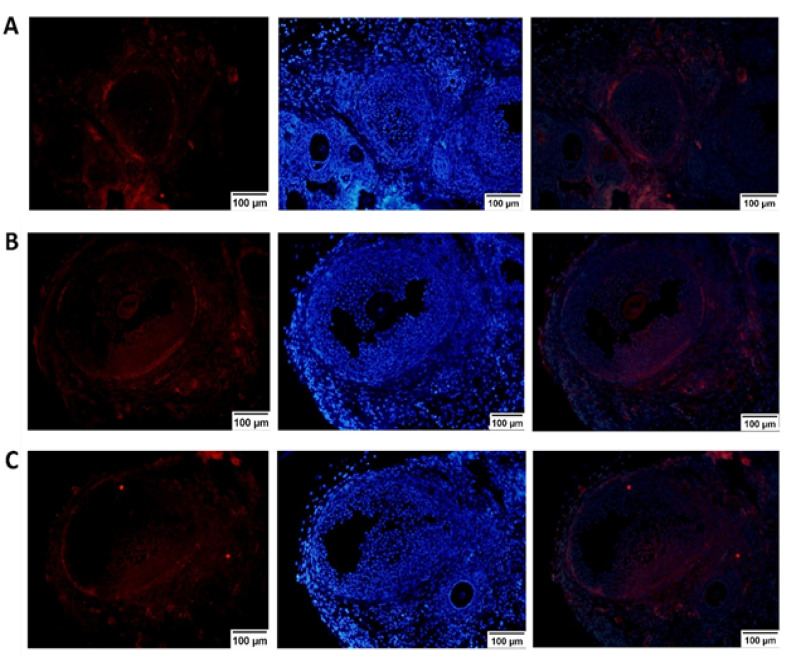
Immunostaining for vascularization of the developing follicle transplants, two weeks after auto-transplantation. Histological slices were stained with antibody against mouse CD31 (PECAM) and DAPI. **A**: Fluorescence microscopy of immunostained histological sections of fresh fibrin-15% PL follicle grafts. **B**: Fluorescence microscopy of immunostained histological sections of vitrified fibrin-15% PL follicle grafts. **C**: Fluorescence microscopy of immunostained histological sections of vitrified fibrin-0% PL follicle grafts.

Neovascularization inside the fibrin hydrogel was evaluated by CD31 immunostaining. As shown in Figure-6, CD31-positive cells were visualized around and inside the fibrin hydrogel in both cryopreserved and non-cryopreserved groups of fibrin-15% PL. Table-1 shows the calculated vessel area (µm^2^) and the ratio of vessel surface area/ total surface area (µm^2^) in all graft types using ImageJ software. As observed, the percentage of vessel surface area in the grafts incorporating PL was considerably higher, compared to PL-less grafts.

## Discussion

The emerging cryopreservation procedures have become a promising approach toward the preservation of fertility among young women receiving chemotherapy and whole-body irradiation [25]. The present study aimed to evaluate a strategy for transplanting cryopreserved ovarian follicles encapsulated within fibrin scaffold supplemented with 15% PL. This approach may represent a step toward the development of fertility-preserving options among cancer patients by reducing the risk of reseeding malignant cells. Cryopreserving preantral follicles, as an alternative to mature oocyte cryopreservation, has some advantages such as greater availability in the ovary, more effective permeation of cryoprotectant agents through follicular suspension, less sensitivity to toxic effects of cryoprotectants and cryoinjury, and their presence in gonads at all ages, as well as enabling their retrieval from pre-pubertal individuals. Also, cryopreservation of mature oocyte may induce zona pellucida hardening, which may result in inhibiting fertilization. Unlike the pieces of cryopreserved ovarian tissue, freeze-thawed follicles yield better survival due to faster vascularization, which were successfully grafted through suspension in fibrin clot [2,26], alginate [2,4,27], and collagen [2], or a combination of these biomaterials [28]. Fibrin scaffold, as a substrate for cell proliferation, has been widely employed for a variety of tissue engineering applications both in vitro and in vivo [29,30]. This biomaterial controls the release of growth factors and generates a continuous path for cellular infiltration between host and graft [31]. Biodegradability of fibrin matrices is a critical parameter for the spatiotemporal control of tissue regeneration [32]. Several studies have examined fibrin scaffold as a cell-embedding material for transplanting follicles [1,33] and ovarian tissues [31]. Recent reports have shown that the transplantation of isolated follicles in a scaffold could be an alternative to cortical fragment transplantation in cancer patients at the risk of ovarian metastasis [34]. Luyckx *et al*. reported the application of fibrin scaffold for transplanting isolated preantral follicles in mice and concluded that fibrin could be a promising matrix for constructing an artificial ovary [1]. In another study, Xu *et al*. demonstrated the effect of fibrin hydrogel on the survival and growth of individually-cultured primary and secondary follicles isolated from rhesus macaque and found that the percentage of growing primary follicles cultured in fibrin was significantly higher than that of alginate matrix. Further, anti-Müllerian hormone and VEGF concentrations, which are positively correlated with follicular growth and oocyte maturation, were significantly higher in primary follicles cultured in fibrin than those in alginate [33]. Furthermore, fibrin was evaluated in combination with other biomaterials for delivering follicles and ovarian tissues. However, few studies reported the use of fibrin–alginate (FA) 3-dimensional (3D) scaffold for in vitro culture of ovarian follicles. The findings indicated that FA could promote follicular survival, maturation, and meiotically competent oocytes [2,28,35-37]. Additionally, fibrin matrices conjugated with various bioactive growth factors could promote tissue morphogenesis and maturation [32]. Based on the results in the present study, a significant decrease was reported in survival rate after auto-transplantation of cryopreserved preantral follicles, compared to the amount in non-cryopreserved control, which is consistent with some other studies [25,38], since the impacts of exposure to cryoprotectants may lead to partial or total loss of follicle-granulosa conjunction, delay in follicle development, and increase in DNA breaks [38]. However, PL increased follicle survival rate and growth in the antral stage significantly, compared to PL-less follicle grafts. PCs including PL, PRP, platelet-rich fibrin, or growth factors alone were confirmed to enhance proliferation, cell survival, and tissue regeneration in cell therapy and tissue engineering [19,39,40]. In a recent study, the ability of PDGF-coated decellularized meniscus scaffold for integrative heal meniscus tears, as the most common knee injury, was examined. Based on the results, the implantation of PDGF treated scaffold led to a significant increase in the expression of PDGF receptor β (PDGFRβ), migration of endogenous meniscus cells to the defect area, and cell proliferation [41]. In addition, Vaquero *et al.* investigated the effect of PRP scaffold on intra-cerebral transplantation of bone marrow stromal cell (BMSCs) as a promising strategy for the treatment of intracerebral hemorrhage. The results indicated that PRP-encapsulated BMSC transplants improved neurologic functions, activation of endogenous neurogenesis, and integration of donor cells in the injured tissue significantly. It is worth noting that PRP could increase the biological activity and viability of BMSCs and improve functional recovery in this type of cell therapy [19]. Also, PCs and the growth factors secreted by platelets increased the vascularization in cell transplantation [42,43]. The CD31 immunostaining results in the present study revealed a considerable increase in angiogenesis in PL containing grafts, compared to the PL-less transplants. Recently, Samberg *et al. *assessed the effect of polyethylene glycol modified (PEGylated) PRP hydrogel on angiogenic potential of adipose-derived stem cells (ASCs) in vitro and in vivo. In this study, the incubation of ASCs with PRP involving high concentrations of platelet for 14 days resulted in increasing ASC proliferation and expression of vascular gene and protein. Further, in vivo studies on injured rat skin revealed that wound treatment using PRP-ASC hydrogel increased neovascularization within eight days, compared to that of the control [42]. In another study, the angiogenic effect of human PL (HPL) was evaluated in inflamed dental pulp-derived stem cells (iDPSCs). Based on the results, the treatment of iDPSCs with 20% HPL could significantly increase the cell viability of the expression of angiogenic genes (ANGPT1, EREG, FGF2, FIGF, VEGF-A, IGF-1, JAG1, NPR2, PLDXC1, and STAB1), adhesion molecules, cytokine-producing genes, and growth factors (FGF-2, MCP-1, PDGF-BB, HGF, and VEGF-A) [43]. In the current study, the cryopreservation of preantral follicles was accomplished using a cryotop device, which was successfully applied for oocyte [44,45], embryos [45,46] and follicles [47] vitrification. In addition, the cryopreservation process could be affected by the type of cryo-device, which enables loading of the least amount of fluid, and accelerates temperature drop throughout the vitrification process [48]. According to Desai *et al.*, different types of cryo-devices can affect antral cavity development, follicular in vitro development, and oocyte maturation [48]. The cryotop technology, as an advanced minimal volume approach, led to more successful births following the cryopreservation of blastocysts and human oocytes, unlike other vitrification techniques [48]. This method was reported as a simple and reliable technique by providing consistent results and minimal variations between operators [44]. It is worth noting that the closed system of the cryotop employed in this study can prevent from establishing direct contact between the sample and liquid nitrogen, unlike open systems. Thus, it can eliminate the potential risks of cross-contamination during vitrification and storage [48].Further, the present methylene blue staining results indicated a survival rate of 91.28% after vitrification and warming by demonstrating the success of cryopreservation process. The finding is in line with the report of Taghavi *et al., *in which a survival rate of 97.56% was obtained after vitrification by the cryotop technology [49]. In another study, Lin *et al.* reported that 82±5% of the primordial follicles were morphologically normal after 5-min equilibration and vitrification in a sucrose-free solution using the cryotop method [50]. Hence, the type of vitrification method may ultimately influence follicle viability, maturation, and accordingly, the outcome of transplantation.

## Conclusion

 In the present study, the results demonstrated that auto-transplantation of isolated preantral follicles encapsulated in fibrin-PL scaffold following cryopreservation could be considered as a promising candidate for fertility preservation. Also, the developed biodegradable scaffold could significantly improve local vascularization, increase follicle survival, and promote its growth to the antral stage. Further, the PL reduced ischemia and follicle loss in the process of transplantation significantly. Furthermore, the findings raised the probability that improving angiogenesis and cell survival in grafted cryopreserved follicles by means of the fibrin-PL scaffold can be regarded as an alternative to restore fertility in premenstrual cancer patients receiving chemo- or radiotherapy. However, longer-term implantation in future studies is required to evaluate the influence of the developed scaffold on survival rate and follicular growth.

## Conflict of Interest

 The authors declare that there are no conflicts of interest.

## Acknowledgment

 We are grateful for the funding support provided by Royan Institute (grant no. 90000083).

**Table 1:** Evaaluation of Vessel Formation Around and Inside the Follicle Grafts After Two Weeks of Auto-Transplantation.

Graft type	Total surface area of vessels (µm^2^)	Total surface area of analyzed section (µm^2^)	Vessel surface area/ total surface area (%)
Fresh follicles+ fibrin-15%PL	102905.3	384353.7	26.774
Vitrified follicles+ fibrin-15%PL	81972.79	384353.7	21.327
Vitrified follicles+ fibrin-0%PL	68628.83	384353.7	17.856
